# Supplementary figures and images for: Adolescent and adult mice use both incremental reinforcement learning and short term memory when learning concurrent stimulus-action associations
Source: PLoS Comput Biol. 2024 Dec 23;20(12):e1012667. doi: 10.1371/journal.pcbi.1012667 (PMC11706416; doi:10.1371/journal.pcbi.1012667)

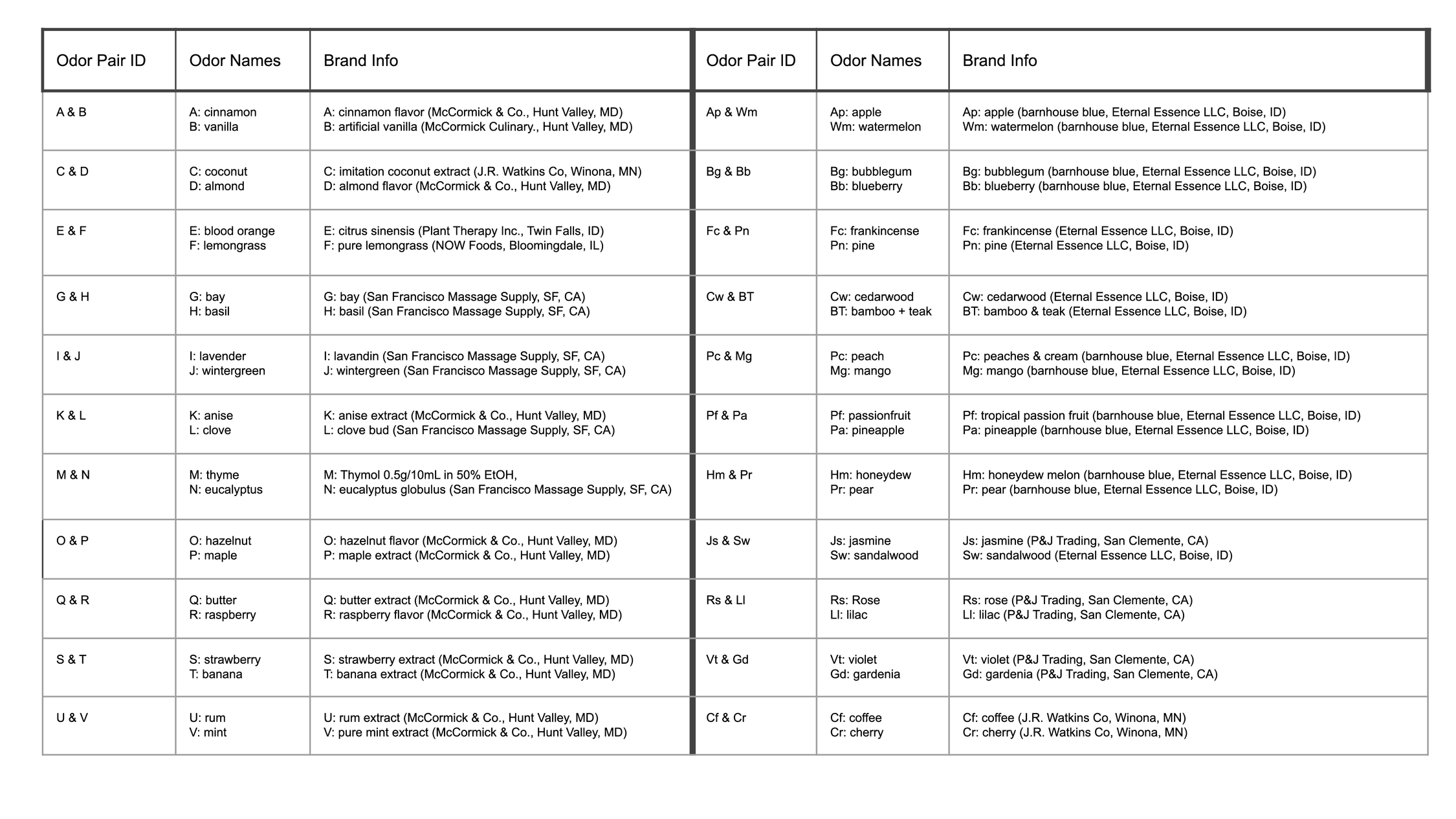

Supplement: S1 Table — Each odor was stably paired with another odor that was similar categorically, but different enough for a mouse to discriminate between the two. With the exception of odors A & B, which was used daily for the “readiness check,” no other odor pairs were repeated. (TIF) [file pcbi.1012667.s001.tif]

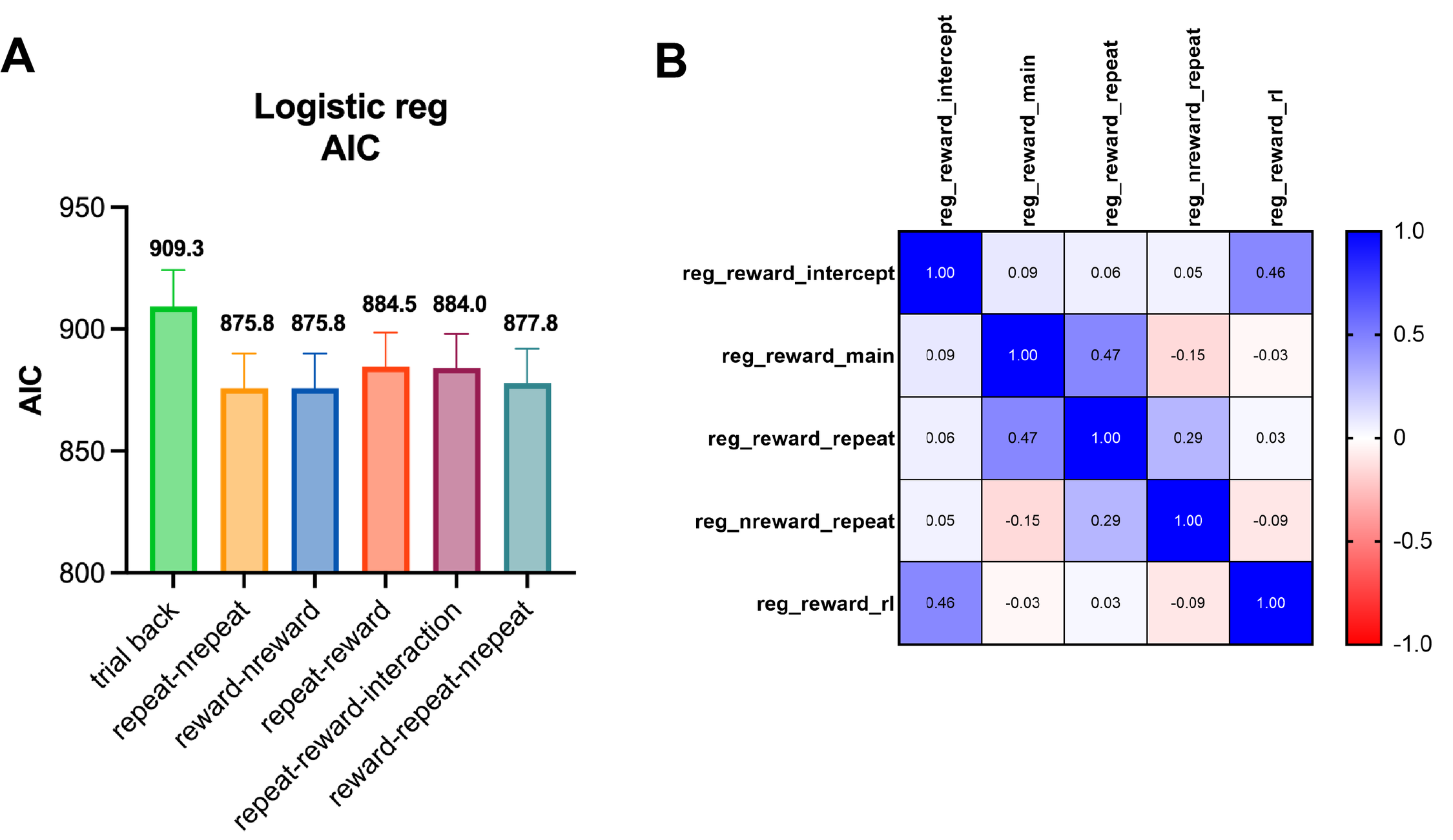

Supplement: S1 Fig — We tested a series of logistic regression models that compared different aspects of task history, trial-back identity, and interactions between various parameters (see Materials and methods for more details). (A) We found that regression #2 (described here as “repeat—no repeat”) and regression #3 (described here as “reward—no reward”) had the lowest AIC and, through additional analyses, determined that the two regressions were mathematically identical. Thus, we chose “reward—no reward” as the winning model and present results for this regression in the main text with regression #2 shown in the supplement. We also checked the correlation between regressors for regression #3 (B) and found that the regression intercept and the correct history parameter (here described as reg_reward_rl) as well as the main effect of reward and the interaction between reward and repeat are both the most correlated. Reward main and reward_repeat draw on the same one-back rewarded information. The negative correlation between no_reward_repeat and both correct history and the main effect of reward aligns with the two outcome choice options in our task design (reward or no reward) which oppose each other. To ensure that correlations do not affect our conclusions, we tested multicollinearity with variance impact factors (VIF) as reported in our Materials and Methods and removed any points outside of 3 standard deviations of the mean (z-scored) before we visualized or analyzed any data. (TIF) [file pcbi.1012667.s002.tif]

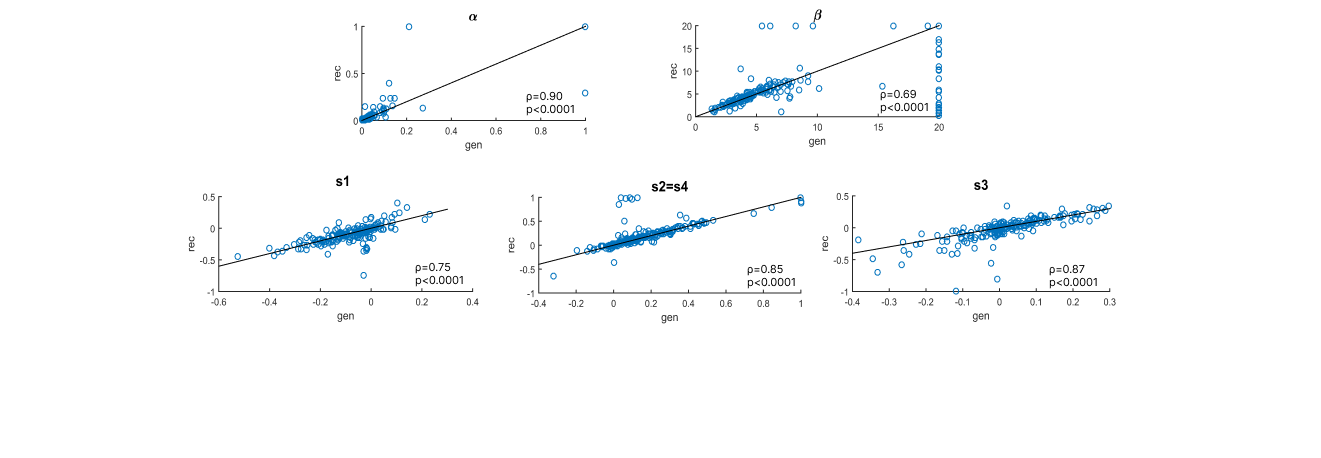

Supplement: S2 Fig — We tested a series of RL-based computational models that explored which strategies mice may use to integrate and use past trial information for trial-by-trial learning. The winning model, as shown by BIC and protected exceedence probability (see Fig 6B and 6C), had 5 free parameters that included α+ (a), fixed α− = 0), a softmax β parameter (b) and 3 one-back strategy parameters (see Fig 6A for schematic, Materials and methods for more details). In order to validate our winning model, we generated data by simulating the model with parameters fit on individual sessions. Then, we fit the simulated data in order to obtain recovered parameters. Recovered model parameters (y-axis) were highly correlated with generated parameters (x-axis; all Spearman ρ > 0.69, p < 10−4), indicating that model parameters are identifiable([29]). Figure corresponds to set size = 2 males, but parameters were equally identifiable for females and set size = 4. (TIF) [file pcbi.1012667.s003.tif]

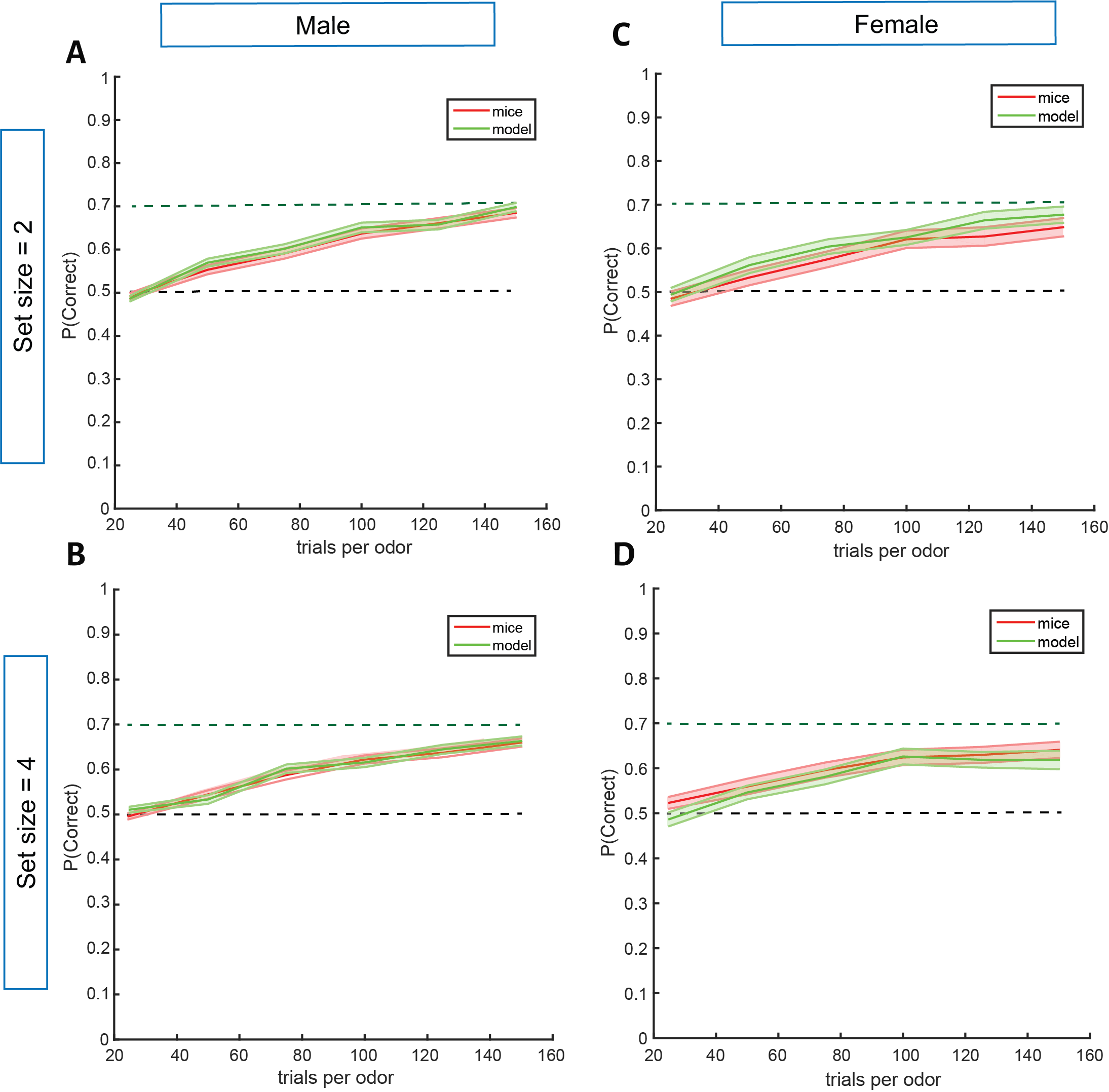

Supplement: S3 Fig — In order for a model to be valid, it is important that it captures both trial-by-trial learning as well as learning that takes place across a session. Our winning model, a0b1232, includes an RL-learning component as described in Materials and methods along with 5 free parameters as described in the name. α+ is indicated by (a), softmax beta parameter by (b), the absence of any α− learning rate is indicated by (0). The final model also included 3 one-back strategy parameters (S1, S2 = S4, S3, see Fig 6A for schematic) that captured how mice’ responses to a current trial reflect one-back stimuli and reward experience. Here, we generated data by simulating the model with parameters fit on individual sessions. Then, we inspected the similarity between simulated data (green) and mouse data (red) and found that the model simulated with fit model parameters captures well mouse learning curve data. All data for males (A-B) and females (C-D) are binned by 25 odor presentations as shown in Fig 2A and 2D and described in Materials and methods. (TIF) [file pcbi.1012667.s004.tif]

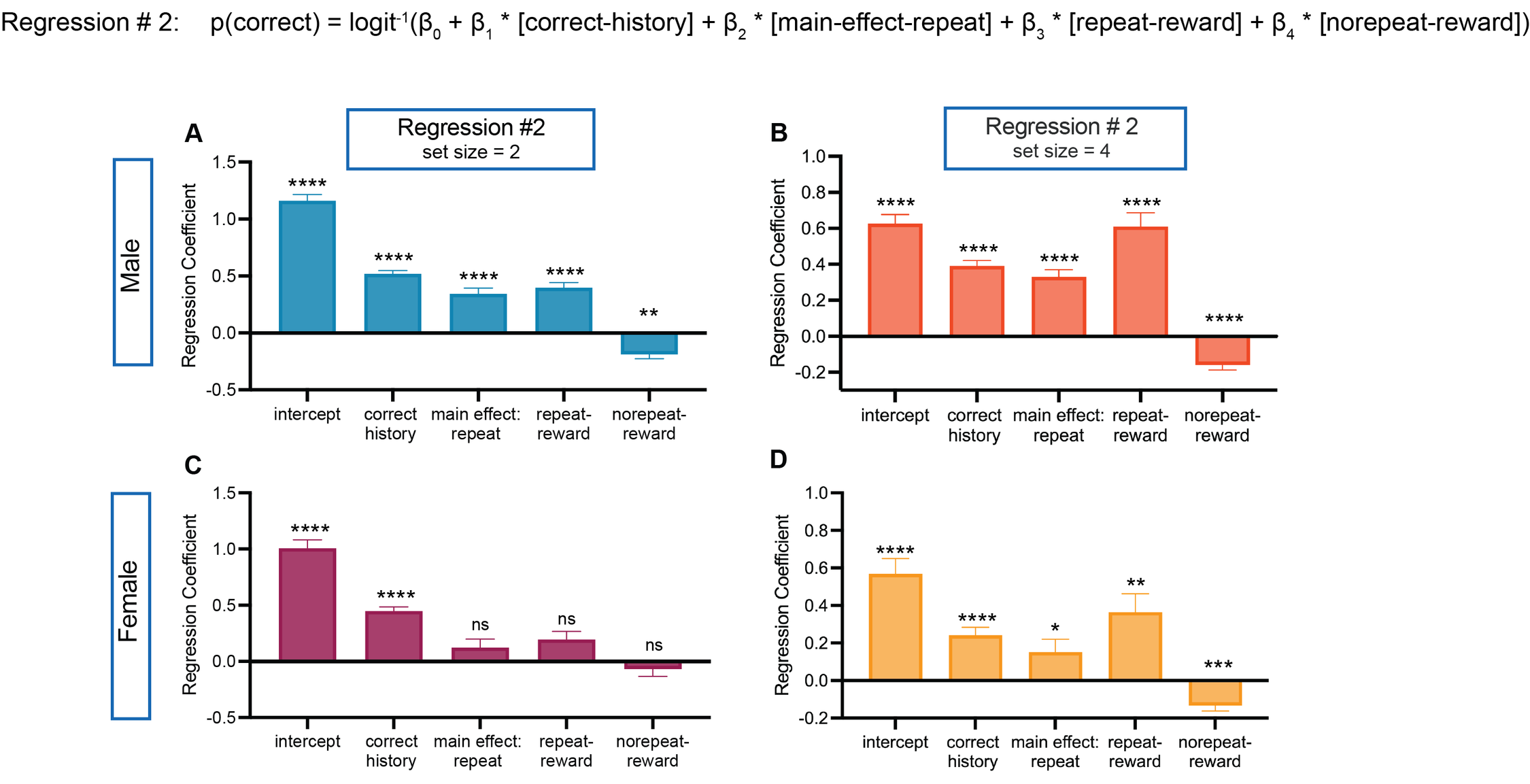

Supplement: S4 Fig — While in modeling space and in AIC comparisons (see S1 Fig) both regression #2 and regression #3 were identical, the separation of the two allowed us to better understand if the main contributor to performance was repeated trials or previously rewarded trials. All bars reflect the mean of all individual session coefficients and error bars represent SEM. For males (A) in set size = 2, there was a significant effect of repeating trials and the interaction between repeating rewarded trials and non-repeating rewarded trials: intercept: t = 17.74, df = 31, p < 0.0001; correct history: t = 14.78, df = 31, p < 0.0001 main effect of repeat: t = 4.7, df = 31, p < 0.0001; repeat—reward: t = 6.59, df = 31, p < 0.0001; no repeat—reward: t = 3.4, df = 31, p = 0.001. For females (C) in set size = 2, there was only a significant effect of repeating trials when the previous trial was rewarded: intercept: t = 10.63, df = 14, p < 0.0001; correct history: t = 14.61, df = 14, p < 0.0001; main effect of repeat: t = 1.16, df = 14, p = 0.26; repeat—reward: t = 1.33, df = 14, p = 0.20; no repeat—reward: t = 0.53, df = 14, p = 0.60. For males in set size = 4 (B), intercept: t = 12.34, df = 31, p < 0.0001; correct history: t = 13.12, df = 31, p < 0.0001; main effect of repeat: t = 8.15, df = 31, p < 0.0001; repeat—reward: t = 8.07, df = 31, p < 0.0001; no repeat—reward: t = 5.62, df = 31, p < 0.0001. Finally, for females in set size = 4 (D), intercept: t = 6.98, df = 13, p < 0.0001; correct history: t = 5.79, df = 13, p < 0.0001; main effect of repeat: t = 2.17, df = 13, p = 0.04; repeat—reward: t = 3.72, df = 13, p = 0.002; no repeat—reward: t = 4.55, df = 13, p = 0.0005. Together these results indicate a significant influence of both correct history (RL) and combinations of one-back trials on current mouse choice. Note that for our second regression for set size = 4, the absolute number of repeat trials are fewer than set size = 2. **** p < 0.0001 *** p < 0.001, ** p < 0.01, * p < 0.05. (T [file pcbi.1012667.s005.tif]

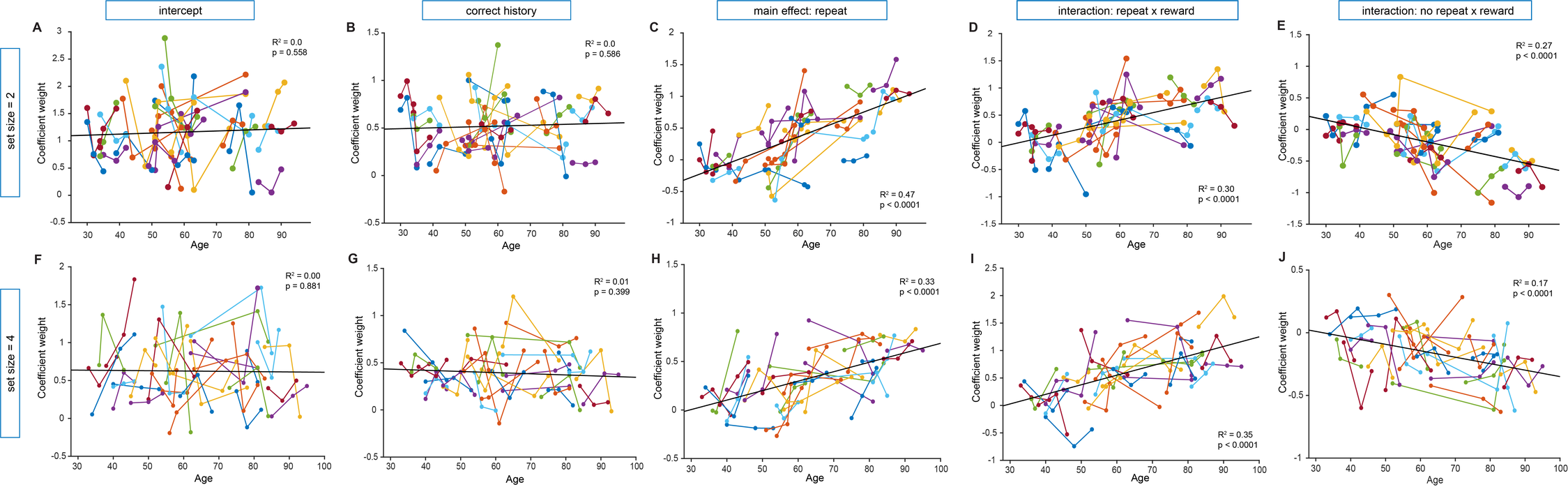

Supplement: S5 Fig — This regression tied with regression #3 for lowest AIC and sets up repeat trials as the main contributor. In order to understand the relationship between age and the predictors of current choice in trial t (see S4 Fig for the logistic regression summary), we looked at whether coefficient weight (y-axis) would change over development (x-axis) for male mice. For set size = 2, (A) repeat intercept: βage = 0.005, 95% CI = [−0.002, 0.01], p = 0.15; (B) repeat correct history: βage = 0.002, 95% CI = [−0.002, 0.005], p = 0.34; (C) main effect of repeat: βage = 0.024, 95% CI = [0.016, 0.033], p < 0.0001; (D) repeat—reward: = 0.014, 95% CI = [0.009, 0.018], p < 0.0001; (E) no repeat—reward: βage = −0.010, 95% CI = [−0.014, −0.006], p < 0.0001. For set size = 4, (F) intercept: βage = 0.00, 95% CI = [−0.006, 0.006], p = 0.994; (G) correct history: βage = 0.002, 95%CI = [−0.004, 0.008], p = 0.561; (H) main effect of repeat: βage = 0.008, 95% CI = [0.005, 0.011], p < 0.0001; and interactions between repeat stimuli and previous reward (I: repeat-reward: βage = 0.011, 95% CI = [0.004, 0.019], p = 0.004; (J) no repeat—reward: βage = −0.005, 95%CI = [−0.008, −0.002], p = 0.001. All developmental changes seen in set size = 4 were also reflected in set size = 2. Statistics and figure structures are identical to Fig 4. (TIF) [file pcbi.1012667.s006.tif]

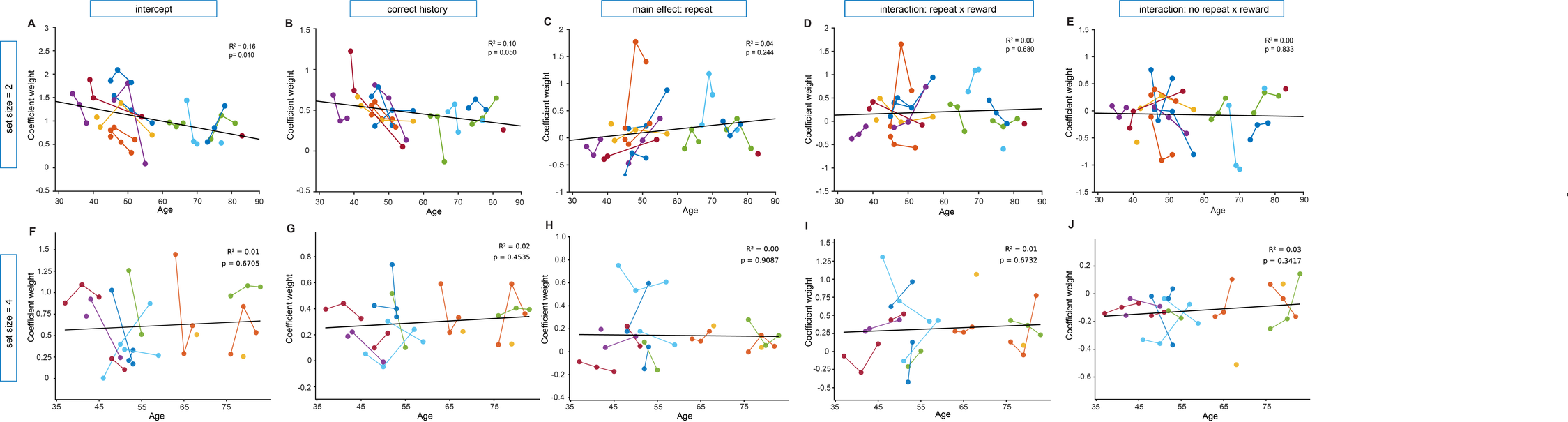

Supplement: S6 Fig — All panels are structured similarly with the same statistical methods as Fig 5. For set size = 2, (B) repeat correct history: βage = −0.004, 95% CI = [−0.01, 0.002], p = 0.20; (C) main effect of repeat: βage = 0.015, 95% CI = [−0.008, 0.039], p = 0.20; (D) repeat—reward: βage = 0.002, 95% CI = [−0.01, 0.014], p = 0.78; (E) no repeat—reward; βage = −0.001, 95% CI = [−0.013, 0.011], p = 0.86. For set size = 4, (F) intercept: βage = 0.002, 95% CI = [−0.001, 0.013], p = 0.797; correct history(G): βage = 0.001, 95% CI = [−0.004, 0.007], p = 0.605; (H) main effect of repeat: βage = −0.001, 95% CI = [−0.009, 0.006], p = 0.735 (I) repeat—reward: βage = 0.003, 95% CI = [−0.011, 0.016], p = 0.683 (J) no repeat—reward: βage = 0.002, 95% CI = [−0.002, 0.006], p = 0.337. While it is possible that a low number of set size = 4 sessions for female mice may be responsible for no trial-by-trial learning changes over development, our findings replicate set size = 2 sessions for female mice. (TIF) [file pcbi.1012667.s007.tif]

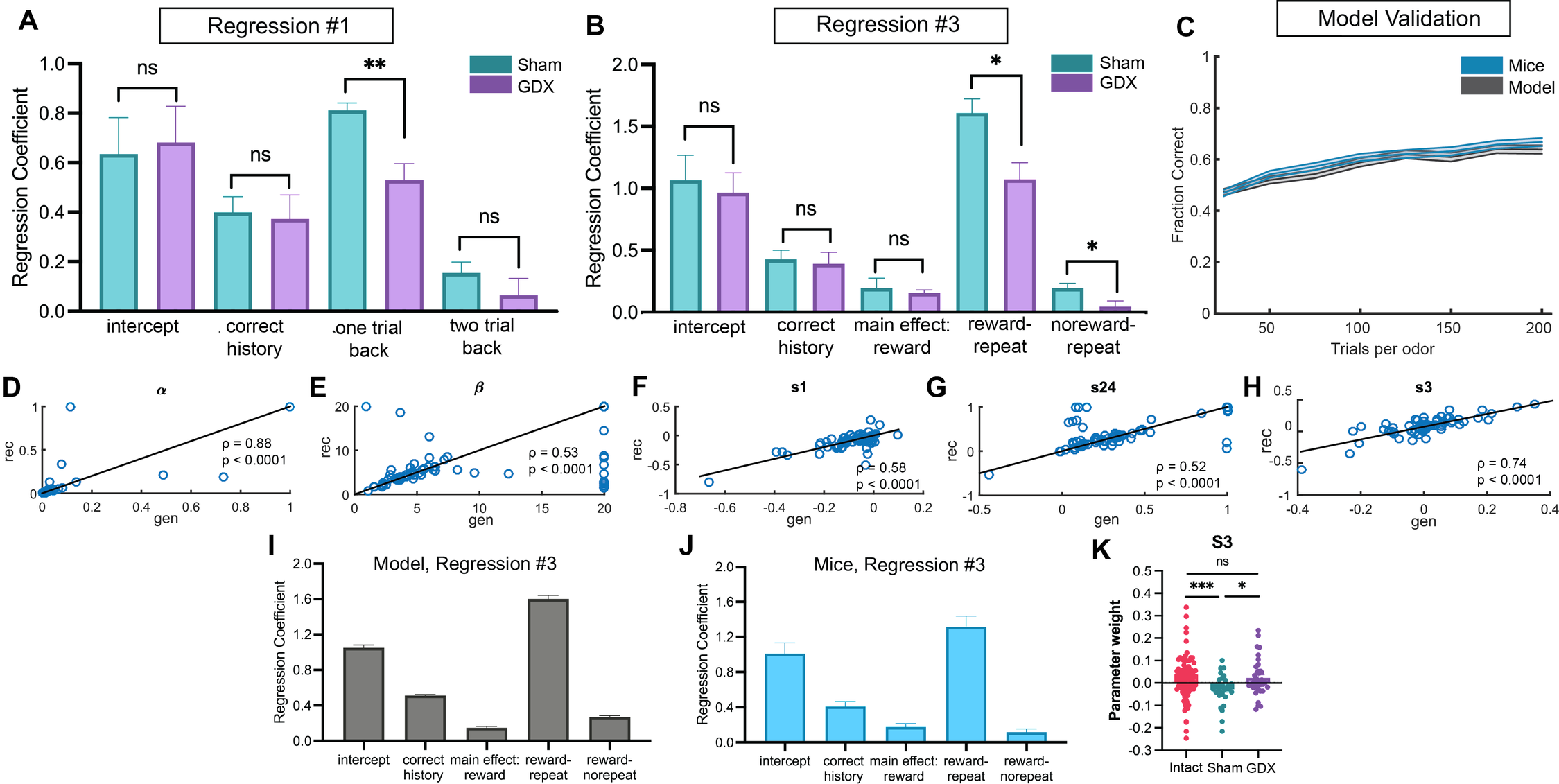

Supplement: S7 Fig — We also fit regression and computational models to the GDX and sham data. For regression #1 (A) and regression #3 (B) GDX (purple) and sham (green) sessions for individual mice for both set size = 2 and set size = 4 were averaged together and the mean of each individual was used to generate the shown group mean with SEM. Both conditions showed comparable regression weights for intercept and correct history across our two main regressions. There were a few differences between groups that reached statistical significance. In regression #1 (A), one-trial back was significantly different between age-matched GDX (n = 6) and sham (n = 5) mice (p = 0.005). In regression #3 (B), both reward-repeat (p = 0.01) and noreward-repeat (p = 0.03) showed differences. These differences in sham and GDX were in the same direction as differences between intact male and female mice for two of these regression coefficients (see Fig 3). However, GDX males were still statistically different from females (set size = 2 and set size = 4 pooled together for comparison to GDX). (For regression #1 (one-trial back), GDX vs. intact females: t = 3.09, df = 33, p = 0.004; mean ± SEM: GDX males: 0.53±0.06, intact females: 0.11±0.05. For regression #3 (reward-repeat), GDX vs. intact female: t = 3.02, df = 33, p = 0.004; mean ± SEM: GDX males: 1.07±0.06, intact females: 0.30±0.05). The last regression coefficient that was significantly different between GDX and sham, noreward-repeat, was comparable to intact female values. These data suggest GDX has subtle effects but does not phenocopy intact females. We next validated our winning model, a0bs1232 by simulating GDX/sham data and running both the simulated and mouse data through the regressions. We show simulated and mouse data for regression #3 in (I) and (J). In (C) we generated data by simulating the model with parameters fit on individual sessions. Then, we inspected the similarity between simulated data (green) and mouse data (red) and found that th [file pcbi.1012667.s008.tif]
