# Supplementary material for: Adolescent and adult mice use both incremental reinforcement learning and short term memory when learning concurrent stimulus-action associations
Source: PLoS Comput Biol. 2024 Dec 23;20(12):e1012667. doi: 10.1371/journal.pcbi.1012667 (PMC11706416; doi:10.1371/journal.pcbi.1012667)
Supplement: Attachment — Submitted filename: ResponseLetter_Chase2024_RLWM.pdf [file pcbi.1012667.s010.pdf]

October 16th

Dear Colleagues,

Thank you for taking the time to review our manuscript *Adolescent and adult mice use both incremental reinforcement learning and short term memory when learning concurrent action associations*. We have made the following major changes to the manuscript in response to the comments.

1. To test if pubertal hormones impact age dependent increases in one back strategies in males, we have added an experimental study of mice with pre-pubertal gonadectomy and sham controls. These data suggest gonadal hormones do not contribute to age dependent increases in one-back strategy use. They also show lack of gonadal testosterone at puberty and in young adulthood has no significant impact on learning, learning rate, and softmax beta parameters, possibly important negative findings.
2. We have revised our presentation of our regression analyses. We have removed some redundancies in the main text and now show more information in the supplement. We have also worked to enhance clarity.
3. We address questions about the computational modeling strategy and methods.

We have also made changes in response to other comments from reviewers (see in line text in black below). We appreciate the opportunity to revise and think that the process has improved our manuscript in terms of content and readability.

Sincerely,

Linda Wilbrecht (on behalf of all authors)

Reviewer #1: The authors were motivated to understand the extent to which working memory (WM) is utilized alongside reinforcement learning (RL) during the acquisition of stimulus-action associations. Additionally, they aimed to investigate whether WM and RL metrics undergo changes during adolescent development. Their research is primarily driven by prior human studies revealing that behavioral performance in simple instrumental tasks is more accurately described by the interplay of two processes. To determine if similar results extend to rodent models, they adapted a task previously used in humans for use with mice. They trained mice at different ages in an odor-based two-alternative forced-choice task, varying the size of the learning problem (i.e., the number of odors to associate with a left or right action) to manipulate WM effects. They conducted preliminary data analysis to inspect possible behavioral signatures of WM. Next, they performed three logistic regression models to assess the contributions of both incremental RL and WM to choice behavior. Based on the regression results, they built different computational models that extend a simple Rescorla-Wagner learning rule to assess their potential in enhancing data fit. These mechanisms include learning rate asymmetry, decay of values over time (forgetting), and the implementation of strategies based on previous information (one-back strategies), including the tendency to repeat the previous action or incorporating information about the trial history. Finally, leveraging the longitudinal nature of their design, the authors examined the effect of age on task and model metrics. Overall, I believe the work addresses a significant topic by validating human paradigms in the murine model, which holds paramount importance due to its genetic manipulability. Nevertheless, this current version of the manuscript has several major issues that the authors need to address.

Reviewer #2: Summary

The authors contribute to computational modeling of behavior by examining the interplay between reinforcement learning (RL) and working memory (WM) in mice performing a 2AFC odor discrimination task using interleaved odor set sizes of either 2 or 4 novel odors. They also examine how utilization of these strategies changes with age, gender, and odor set size. Their goal was to establish a comparative baseline between humans and mice when measuring RL+WM, and whether mice show similar strategy changes between set sizes and throughout development. Using two modeling approaches, they find that mice employ both RL and a simple form of WM captured as 1-trial-back perseveration dependent on previous odor and reward, without finding significant differences between odor set sizes. They report age-related changes in the utilization of WM, but these changes are inconsistent across male and female mice. They don't find age-related changes in RL in either gender. These results contrast with findings in humans, highlighting differences between the two species.

Overall, I find the work to be an important contribution towards understanding species differences in the deployment of RL vs WM strategies, especially when mouse models are frequently used as a proxy to study similar mechanisms in humans. However, the modeling of RL alongside other strategies is not itself a novel contribution (e.g. Miller et al., 2017, Nature Neuroscience; Akam et al., 2021, Neuron; Miller et al., 2022, eLife; and Venditto et al., 2024, eLife, all model strategy mixtures combining RL with perseverative agents similar to the RL+WM model used here); although the specific setting in which the model is applied here appears novel. Additionally, their specific parameterization raises some concern over how its interpreted (major issue below). The manuscript would also benefit from additional clarification and justification (minor issues below).

We thank the reviewers for their time and supportive comments.

Major issues

• RL+WM model parameterization: Although the equation before line 190 is missing a closing parenthesis, given the equation before 188, it is fair to assume that the closing parenthesis should be at the end. In both equations, then, the softmax beta acts on both the RL Q(o,a) as well as the choice kernel I(a,at-1). In this parameterization, beta acts as a decision noise parameter on all choice-related information and should not be interpreted as a weight on the RL component. This raises concern over the interpretation of data in figures 7B,G, 8B,G, and 9B,G, which does not fully represent changes in RL strategy across age or sessions. Furthermore, in all these figures I find the log-scale to be slightly misleading towards the true variation in beta weights, which seems strikingly large. Many of the weights seem to hit a ceiling at log(3)=20, which also appears to be an issue in the parameter recovery data shown in figure S1, suggesting that these fitted values are unreliable. It is possible that parameter interactions between beta and the WM weights are contributing to this fitted behavior. Another contributor may be the lack of modeling a bias (Wilson & Collins 2019).

Answer: Thank you for flagging the missing parenthesis - the reviewer's assumption about its location is accurate, beta does act on both RL and I. Note that none of our conclusions are focused on the beta parameter, which is the one where issues are flagged here. We address the multiple comments in this point below.

- Interpretation of beta parameter. We fully agree with the reviewer that, for this reason, beta cannot be interpreted as a purely RL parameter; we have corrected language in the manuscript that suggested this (including caption of fig. 7 and 8).
- Fitting issues. We agree that some fit beta parameters hit the upper bound we imposed as a constraint in the fitting. This is a common issue due to the fact that the likelihood surface is often extremely flat within high beta values, with increases in beta improving the likelihood in extremely small amounts that are non meaningful, but sufficient to move the optimizer to the bound. This is typically handled with adding a prior on the parameter. We chose here not to do this for a few reasons: 1) we obtain generally good parameter recovery for beta (as evidenced in Fig. S2), and this issue is limited to a few data points 2) adding a prior requires additional choices (prior hyperparameters) that add analysis degrees of freedom; 3) we do not draw strong conclusions on individual beta parameters, such that strong confidence in beta recovery is not necessary; 4) We use non parametric tests where possible to handle the non-normal parameter distribution due to the boundary.
- Plotting issue. We choose to plot beta as log(beta) for two reasons. First, it is a monotonic transform, so it still enables comparison between groups. Second, when large beta values are within the parameters, their impact on behavior is decreasing (see point above); this makes log(beta) a reasonable visualization of the impact of beta on behavior. Overall, we think that, given our transparency in indicating what is plotted, this is a reasonable visualization choice.

Minor issues

• Materials and methods: Behavioral task: there are some details missing that inhibit replicability, as well as some things that need further clarification

- What is the total number of odors used, and what odors were they?
- Were any odors reused outside the preliminary odor pair, or was it a novel set in every new session?
- How were odor set sizes interleaved?
- On line 83-84, does "six sessions total per mouse" include the two to three training sessions, or does this mean three sessions of each set size, leading to six sessions in addition to early learning?
- It might be helpful in Figure 1 to give an example of odor sequences presented within a single session to clearly lay out the A/B odor phase and transition to novel odor set

• Results: Behavioral task and results: While a second description of the task is useful in this section, including details of pre-training feels redundant as it doesn't have as much bearing on the results.

Answer: We apologize that these details were not more clear. We added additional information into the behavioral task and statistics description (see Materials & Methods), including a table that describes the pairs of odors used (see Supporting Information Table 1, S1 Table). Outside of the preliminary odor pair (always odors A & B which became our "readiness check"), no odor pairs or single odors from the odor pairs were reused. Odor pairs were paired due to their similarity (e.g. fruits were always together) and stayed consistent for all mice. Most mice started on odors C & D as set size = 2 and progressed through the alphabet in order, but due to limitations on the number of odor filters for each individual odor, a minority of mice were started on odors E & F or G & H instead and progressed through the series of odor pairs from that starting point. In additional analysis on a separate cohort, we've determined that mice learn similarly across odor pairs (looking at binned or quartile learning curve data across 7 odor pairs, unpublished data). The majority of the male and female mice experienced set size = 2 prior to set size = 4, with most mice experiencing two set size = 2 sessions prior to set size = 4. Because some mice had different set size ordering, we chose to examine model parameters across age and then to also examine the effect of session not separated by set size.

Here is the updated information from our Materials & Methods: "For pre-training, mice were water-restricted and then trained to initiate trials via nose poke to the center port and to associate side ports with water reward for several days. Next, mice entered an early learning phase where they encountered a preliminary odor pair, A(cinnamon) - B(vanilla), which stably predicted the left and right port respectively for reward. These odors were presented for two or three days. Next, mice entered the main task where they learned to associate novel odor pairs to a port, using deterministic reward outcomes. Mice experienced a novel odor daily if they passed a "performance readiness check" by reaching performance levels of 70% on both sides for odor pair A&B at the start of each session. In our experience, this check primes mice to use an odor-based strategy. Mice were run for at least six sessions with novel odors presented in set sizes of n=2 or n=4 (a minimum of three sessions per set size) for the full RL+WM task. See Supporting Information Table S1 for a list of all odor pairs used (Table S1). If mice failed to reach this performance readiness criterion they were only exposed to odor A&B for that session. Each full task session, one per day, included 100-200 presentations of odors A&B followed by 200-400 presentations (dependent on set size) of each novel odor in a pseudo-random manner, controlling for a normal delay distribution between two successive presentations of the same stimulus. All mice experienced set size = 2 prior to set size = 4, with most mice experiencing two or more sessions of set size = 2 first. Outside of daily exposure to A&B no single odor or odor pair was repeated. "

Based on this suggestion, we updated our first figure with panel B to include a clearer representation of what a mouse experiences within each session and clarified the task description in the methods while streamlining the description in the results section of the text.

• Logistic regression analysis and models: I feel the selection of predictors for regressions 2 and 3 could be better motivated, and the benefit of splitting it out this way is unclear. Neither regression allow for direct comparison between main effects of cues and rewards (as would p ~ 1 + repeat + reward + repeat-reward), nor do they allow direct comparison of possible interactions (as would p ~ 1 + repeat-reward + norepeat-reward + repeat-noreward). They're also technically the same model, where one can be written as a linear combination of the other (e.g. reward = repeat-reward + norepeat-reward; noreward-repeat = repeat – repeat-reward). This also means the coefficients on correct history should be identical across the two models, which appears to be the case in Figure 3, Figure S3 (despite different scales), Figure 4B,G, Figure S4B,G, Figure 5B,G, and Figure S5B,G. These panels should not be treated as separate results.

- In methods lines 116-117, the justification is "to capture different hypotheses", but doesn't explain what those hypotheses are. Also, both regressions 2 and 3 should be outlined here, not just 3.

- In results lines 297-298, the justification is to "triangulate the potential interaction between repeated cues and reward", but neither model allow for direct comparison between main effects of cues and rewards nor all interactions.

- The Figure 3 caption states that regression 2 captures "the influence of stimulus repetition" and 3 captures the "influence of past trial's reward", but it's unclear how these models separate the two when both models include past influence of both stimulus and reward, just with a different parameterization.

Answer: we apologize for lack of clarity in the regression analyses. We initially performed a total of six regression models that included various combinations and interactions of repeated and rewarded trials. We had not included all the details of our additional regressions in our initial draft; but, because of your and another reviewer suggestions, we have now placed a complete description of each into the methods, highlighted only regression 3 in the main text with regression 2 in the supplement (see below for more details) and included a supplemental figure (Fig. S1) showing the results of an AIC comparison across all 6 regressions we tested.

Below is a short description of our 6 regressions (now also included in Materials and Methods):

- Regression #1: correct/reward ~ intercept + correct history + 1 trial back + 2 trial back.
- Regression #2: correct/reward ~ intercept + correct history + main repeat + repeat-reward + norepeat-reward (where rewarded = 1, not rewarded = -1).
- Regression #3: correct/reward ~ intercept + correct history + main reward + reward-repeat +

noreward-repeat (where repeated = 1, not repeated = -1).

- Regression #4: correct/reward ~ intercept + correct history+ repeat-reward + repeat-noreward + norepeat-reward + norepeat-noreward (where each interaction term is either 0 or 1).
- Regression #5: correct/reward ~ intercept + correct history + main repeat + main reward + reward*repeat.
- Regression #6: correct/reward ~ intercept + correct history + main reward + main repeat + repeat-reward + nrepeat-reward (where interaction term rewarded = 1, not rewarded = -1).

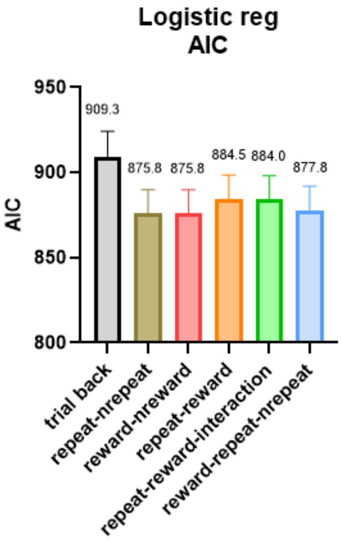

Model comparison: Using AIC comparison (as well as AICc, CAIC, and BIC), regression #2 and #3 are better models than regressions 1,4,5,6 (see included figure and now supporting information Figure S1 in our manuscript).

Regression #2 and #3: In our winning regressions, we looked at the main effects of repeat or reward separately and also looked at how each main effect interacted with the other factor. For example, in regression #2 (correct/reward ~ intercept + repeat + repeat-reward + norepeat-reward), both rewarded and not rewarded were bidirectionally coded in the term repeat-reward and norepeat-reward, where rewarded = 1, not rewarded = -1, accounting for all possible interactions between repeat and reward.
We separated regression #2 and #3 under 2 angles, where regression #2 examined repeat odor presentation as driving the main behavioral differences while effects of reward were captured in interaction terms. Regression #3 examined previous rewards while the effects of repeated odors were minor and captured in interaction terms. These are now discussed in our Materials and Methods section.

In modeling space, regression #2 and #3 contain terms that are interchangeable using linear combination computation, and are thus mathematically equivalent. Despite this, performing regressions separately allowed us to examine the results through different angles and to interpret effects of interactions accordingly.

• Materials and methods: One-back strategies: Although it was clarified in the results, the mapping between trial type (repeat or non-repeat, rewarded or unrewarded) to trial number (1-4) is laid out in the methods. Therefore, taking this section by itself, it's unclear why setting s2=s4 is a valid pairing.

Answer: We apologize that this portion of the methods was unclear.

We have added an additional couple sentences to the methods section one-back strategies: "We considered various models with subsets of those policies set to 0 or to each other's values (e.g. s2=s4) in order to simplify the model complexity. In these analyses we found that parameters s and s4 were highly correlated when fit separately, prompting us to investigate a model where they are set equal to each other, which resulted in our winning model (s2=s4)."

This was to help clarify that we tested correlations between all strategy parameters and found that *s2* and *s4* were highly correlated. Given this, we fixed the parameters as equal to each other and our winning model contained 3 strategy parameters, instead of the original 4.

• Results: Computational Modeling: The name associated with S1, "Inappropriate Lose-Shift", appears confusing because, by construction, all one-back strategies should reinforce making the same choice. Shouldn't S1 bias the policy towards repeating choices after a stimulus change and previously unrewarded choice? Or has the weight on S1 been flipped prior to plotting to correspond with choice switching?

Answer: We apologize for any confusion with the naming of our strategy parameters. Each strategy parameter (S1: "Inappropriate Lose-Shift", S2: "Inappropriate Win-Stay") is named for the action associated with positive parameter value. To clarify this we have added language to our legends to help with parameter interpretation and we also enlarged components of the 6A parameter schematic to better assist with understanding our model.

We also understand that S1 is the only strategy parameter where the same choice is not reinforced and understand any confusion that that creates. This, unfortunately, is linked to decisions made early on in the model development process that are difficult to change without restructuring and redoing much of our analyses.

• Figures: For many of the bar plots, it is unclear from the figure legend what is being plotted (mean or median), what the error bars are computing (e.g. SEM, 95% CI), and what each are being computed over (rats or sessions). Some figure specific comments:

- Figure 3,S3: it would be useful to have asterisks to visually identify significant bars

- Figure 4,5,7,8: Putting asterisks next to significant p-values would help identify significant regressions

- Figure 6C,D: Make explicit what "exp_r" and "pxp" is in the figure legend

- Figure 7: What is the red box around panels D and I?

- Figure S1: What are the correlation coefficients for each panel? It would be useful to have this information overlaid on each plot

• Line 117: "logistic link function" should be "logit link function"

• LaTeX formatting: Most of the opening quotation marks are backwards. There are also redundant parentheses around in-text citations.

Answer: We appreciate your attention to detail on the text and figures and your suggestions to clarify our results and reporting. We changed "logistic link function" to "logit link function" and corrected quotation marks and redundant parentheses. We added more information for the summary regression figures legend (clarification on error bars, what is being computed, etc) and added asterisks to summary regression figures that correspond with the p-value significance. We also clarified across all figure legends which stats were performed and what error bars indicate. We had initially placed a red box on

Figure 7D,I to show the similarity across set sizes for male animals, but have removed it in favor of bolding all p-values that are significant and for streamlining down the legends to only include the significant p-values that were measured. We have included additional sentences in the Figure 6 legend to clarify what "exp_r" and "pxp" references:

"However, a Bayesian model comparison analysis showed that a0bs1232 was significantly more frequent across sessions (C; posterior frequency 39.74% vs. 26.11% for a0bs1234), with a protected exceedance probability (pxp, (Rigoux et al., 2014)) of 0.991"

We have also re-run the gen/rec figure and added correlation coefficients for each panel (it is now Fig. S2).

Reviewer #3: Chase and colleagues present a study investigating the use and development of different learning strategies from adolescence to adulthood in mice. Adapting a task developed in humans that can be solved, to different extents, by reinforcement learning and working-memory based mechanisms mice learned odor-based conditional associations in small or large set sizes. The authors analysed the data at the gross performance level, as well as using regression analyses and computational models. The results suggest, contrary to humans, that mice rely more on RL strategies, with little evidence of a clear WM influence on task performance. While there was evidence that male mice performed better when there were fewer items to learn this was related to an increased reliance on repeating a choice after a reward (win-stay), which was both more likely to happen in the smaller set size, and increased across age.

As the paper stands I believe it could make a valuable contribution to the literature and the justification behind the work in providing a greater knowledge of the value of mice models to understanding some aspects human cognition and cognitive dysfunction is hugely important. I believe the study design was a pragmatic adaptation from the human work for use in mice, and the analyses are comprehensive and carefully applied.

Answer: We appreciate your supportive comments.

However, my review focuses on the interpretation of the results in the context of the challenges in translating tasks between humans and non-human animals. While a direct answer to these points is not possible without conducting additional experiments (which I don't necessarily suggest), the results of such future work could reduce our confidence in the current paper's conclusions by challenging the results.

For example, the inclusion of the readiness check requires the animal to complete up to 200 trials before the main experiment. The authors, already point to this as a difference between the present study and other past work that suggest mice can use WM-based strategies. However, the authors could do more to explore the potential mechanisms that this difference may affect and the impact of that specifically on their current result. One potential difference the readiness check introduces is fatigue which likely reduces WM capacity. Another would be motivation, as by the time the mice reach the main

experiment they will have relatively more of their daily water allowance compared to mice in other experiments. While these internal states may seem minor experimental differences they may shift the balance of influence of different learning strategies, making it appear that mice are less able in this context to use WM-based mechanisms. I think this weakness could be more clearly considered in the discussion.

Answer: We appreciate your concerns and this chance to consider more deeply the effect of our task design. We now discuss the readiness check and our motivation to use it more clearly. We also argue from data why we think fatigue and demotivation were not an issue, pointing out that we use only the first 200 trials for each odor and mice work for far longer after that.

“Another design aspect of our experiment that may be an important caveat for comparisons to others' data is the "readiness check." We started each daily session by having mice perform the task with odors A&B, only moving them on to learning novel odors when they passed a performance criterion (see Materials & Methods). We chose this design to increase the chance that mice would be engaged in learning about the novel odors, given they had successfully, if briefly, indicated they were able to perform that day using task rules. However, this "readiness check" filters out participation on days with a less efficient state for learning, which may be included in other studies which do not use this method. We do not think the readiness check reduced motivation in the main task because after mice passed the readiness check they continued on to perform hundreds of additional trials per odor beyond the first 200 trials per odor that we studied here (males: 914 mean total novel odor trials, 207.18 SD; females: 987.36 mean total novel odor trials, 213.35 SD).

We would argue the readiness check was unlikely to reduce WM use, because it encouraged stimulus sensitivity. Some past rodent tasks measuring WM also required the animal to pass more than a hundred trials (Liu et al., 2014) and still reported WM.”

Similarly, the differences between male and female mice are intriguing and could be discussed in greater detail. The current interpretation that the increase in win stay strategy was related to the greater energy cost in moving males’ heavier bodies feels very speculative. If the authors feel strongly about this point, I would appreciate more evidence to link these two things together.

On a related point, a potential unexplored methodological difference that may explain the lack of developmental effects in the females is that puberty is not considered in this study. There is evidence that female mice, like humans, go through puberty at an earlier age than males. It is therefore possible that the key developmental window was missed in females in this study. While this would not explain the overall male/female difference a developmental shift still may occur in both species. I suggest this potential issue could be noted in the discussion.

Answer: We agree that the sex difference observed in our study is intriguing and were also interested in the effect that puberty may have on performance and learning strategy differences. Our labs and others have shown that pubertal hormones have an organizational effect on brain areas/circuits that may be recruited for learning and memory (Schulz & Sisk et al., 2020; Delevich et al., 2021). Specifically, past work in the Wilbrecht lab showed that maturation of pyramidal neurons in the dorsomedial prefrontal cortex (dmPFC) required intact gonads in male mice (Delevich et al., 2020). In the Collins lab, using the task with human participants that inspired our study, adolescent human participants were shown to be more affected by set size and have lower performance than adults (Master et al., 2020). By measuring

testosterone and puberty developmental score, they found that WM decay and capacity was affected by pubertal score and testosterone levels, respectively (Master et al., 2020).

With this in mind, we now include additional data from young adult male mice (tested age 61 to 96) with prepubertal gonadectomy and sham surgery as controls (n = 6 for GDX, n = 5 for sham) (see Figure 10 and Supplemental Figure 7). In these experiments, we found no difference in learning between groups and only a single minimal difference between GDX and sham mice in a single one back strategy, parameter S3.

We did not measure estrous timing or ovarian hormones due to time and fund limitations. It is possible that individual female differences associated with cycle were diminished when averaged together. We admit that prepubertal GDX in males only is somewhat unbalanced, but we hope that the imbalance can be tolerated as it addresses the age effects seen exclusively in males.

A final, very minor point is that I believe that many of the figure legends contain too much detail and may be repetitive. Its unusual to include a lot of stats results in main text figures, particularly if they overlap with what's included in the manuscript body. Furthermore, the structure of the figures repeats between male and female so the repetitive summaries could be shorted to "figure format is identical to the males", for example.

Answer: We appreciate your feedback on the density of the figure legends and have addressed this throughout our manuscript by dramatically cutting back on the redundant statistic reporting and the redundant phrasing between figures that are identical except for sex. We kept the significant p-values in the legends so that the important information could still be easily ascertained.

From Email Attachment:

1) The authors need to better justify their task design. Why was each stimulus uniquely associated with one action? This choice may have led the mice to infer the correct action for certain odors based on knowledge of the correct action for other odors, thereby potentially reducing the implementation of both RL and WM This is a possibility also considering that before being introduced to novel odor sets mice started the session with the A/B task to which they were overtrained. In other words, the presentation of the AB task could have served as an initial recall of the task rule (Odor X = Left,Odor Y the opposite), increasing the likelihood that the mouse would utilize a "by-elimination" strategy in the subsequent block.

Answer: Thank you for raising this point. We designed our task to closely mimic the human task used in Master et al. (2020) and related work by Anne Collins to specifically test whether mice exhibit human-like working memory (WM) and reinforcement learning (RL). In these human studies, each stimulus is uniquely associated with one action in low set sizes, allowing for a clear test of the participants' ability to hold associations in WM and then, as stimuli numbers increase and the action set stays stable, use RL over time. We aimed to replicate this design in mice to evaluate whether they would show similar reliance on WM when faced with a fixed, rule-based task and whether, once WM reached capacity, they would shift to another strategy.

We believe that testing WM in a probabilistic task (which we believe you may be suggesting), where stimuli are sometimes associated with different outcomes (e.g., sometimes left, sometimes right), would confound the animals' ability to learn and maintain task rules in WM. This design could shift the task to being too dependent on probabilistic RL rather than a direct WM test. Additionally, our goal was to assess set size effects, particularly using set size = 4, which we believe places a heavier load on WM. These larger set sizes allow us to probe WM capacity more effectively, as seen in the human studies, while keeping task structure consistent. While we believe the mice are using a more sophisticated strategy where they learn both pairs simultaneously, utilization of a "by-elimination" strategy in low set sizes is, in itself, a form of WM that holds in WM at least one stimulus-action association, ensuring that animals are less likely to use a one-side only or a random strategy that totally bypass either WM or RL.

Regarding the possibility that the A & B "readiness check" could serve as an initial recall of the task rule, it is true that the animals are overtrained on A & B before encountering novel odors. However, we deliberately structured the A & B component to act as a performance readiness check rather than an explicit reinforcement of the learning strategy. As you suggest, the A & B task may prime the mice to recall the rule (Odor X = Left, Odor Y = Right), but we consider this advantageous in allowing them to stabilize performance before introducing the novel odor sets. This ensures that variability in performance reflects their ability to manage novel stimuli rather than the challenge of recalling the overall task rule. Importantly, in the set size = 4 condition, the increased cognitive load likely reduces the effectiveness of an "elimination" strategy, encouraging reliance on both WM and RL systems.

2) Further it is not specified how many new odors the author used in total and how did they manage block presentation ordering. All this information should be in the Materials and Methods section.

Answer: Thank you for the suggestion and we apologize that this information was not included in the first draft. We have now included much more information in the Materials and Methods section as well as a table in our supporting information (Table S1) that lists all odor pairs that we used in the task and how they were paired. The majority of the male and female mice experienced set size = 2 prior to set size = 4, with most mice experiencing two set size = 2 sessions prior to set size = 4. Because some mice had different set size ordering, we chose to examine model parameters across age and then to also examine the effect of session not separated by set size.

Here is more complete information from our Materials & Methods:
"For pre-training, mice were water-restricted and then trained to initiate trials via nose poke to the center port and to associate side ports with water reward for several days. Next, mice entered an early learning phase where they encountered a preliminary odor pair, A(cinnamon) - B(vanilla), which stably predicted the left and right port respectively for reward. These odors were presented for two or three days. Next, mice entered the main task where they learned to associate novel odor pairs to a port, using deterministic reward outcomes. Mice experienced a novel odor daily if they passed a "performance readiness check" by reaching performance levels of 70% on both sides for odor pair A&B at the start of each session. In our experience, this check primes mice to use an odor-based strategy. Mice were run for at least six sessions with novel odors presented in set sizes of n=2 or n=4 (a minimum of three sessions per set size) for the full RL+WM task. See Supporting Information Table S1 for a list of all odor pairs used (Table S1). If mice failed to reach this performance readiness criterion they were only

exposed to odor A&B for that session. Each full task session, one per day, included 100-200 presentations of odors A&B followed by 200-400 presentations (dependent on set size) of each novel odor in a pseudo-random manner, controlling for a normal delay distribution between two successive presentations of the same stimulus. All mice experienced set size = 2 prior to set size = 4, with most mice experiencing two or more sessions of set size = 2 first. Outside of daily exposure to A&B no single odor or odor pair was repeated. "

3) The rationale behind the inclusion of the readiness check should be better justified, considering that the motivational aspect could have been controlled post-hoc discarding sessions in which mice did not reach the minimum number of trials performed to be considered engaged in the task.

Answer: Thanks for the comment. We have now expanded our justification of the readiness check in the discussion. While post-hoc discarding of unengaged sessions was an option in early design, we opted not to do this as we feared mice might begin to rely solely on stimulus-insensitive strategies if novel odors were presented daily and success was rare.

We can in more words here explain that the readiness check can ensure that animals are competent at performing the task under familiar conditions before progressing to novel odors. This success might mean that we can have more confidence that variability in performance during the novel odor sessions is more likely to reflect the learning of new associations, rather than engagement/motivation issues or 'misunderstanding' of the task.

Regarding motivation, mice complete hundreds to a thousand + trials in the novel odor conditions following the readiness check, suggesting they remain motivated and do not experience significant fatigue or satiation. we would be more concerned if it was a challenge to get 200 trials per odor after the readiness check but this was not an issue.

Edits to Discussion: ""Another design aspect of our experiment that may be an important caveat for comparisons to others' data is the "readiness check." We started each daily session by having mice perform the task with odors A&B, only moving them on to learning novel odors when they passed a performance criterion (see Materials & Methods). We chose this design to increase the chance that mice would be engaged in learning about the novel odors, given they had successfully, if briefly, indicated they were able to perform that day using task rules. However, this "readiness check" filters out participation on days with a less efficient state for learning, which may be included in other studies which do not use this method. We do not think the readiness check reduced motivation in the main task because after mice passed the readiness check they continued on to perform hundreds of additional trials per odor beyond the first 200 trials per odor that we studied here (males: 914 mean total novel odor trials, 207.18 SD; females: 987.36 mean total novel odor trials, 213.35 SD).

We would argue the readiness check was unlikely to reduce WM use, because it encouraged stimulus sensitivity. Some past rodent tasks measuring WM also required the animal to pass more than a hundred trials (Liu et al., 2014) and still reported WM."

4) Material and Methods:

line 95-112 : I strongly recommend to re-organize the entire section. Considering the difficulty, I experienced in understanding what was done in figure 1, I think this section should include a description of all the dependent variables that the author analyzed to preliminarily inspect behavior (and how they were computed), and the rational for the analysis.

5) Figure 1 A: It seems that the authors present a learning curve where consecutive trials over the first 200 presentation for each stimulus were divided into 8 bins, and performance was averaged within each bin, is the interpretation correct?

I cannot find an explicit reference in the text regarding how the learning curve was calculated, and consequently, how the analysis for evaluating the set size effect was conducted. The set size effect in male mice appears to be so weak that I wonder if it remains significant across the entire curve (i.e., non-binned). If the data were binned the authors should specificized why and how. The authors should show the raw learning curve at least (starting from repetition 1).

Answer: We took this suggestion seriously and have made substantial revisions to the Materials and Methods section to better reflect the methods used in Figure 2 (we believe you were referring to Figure 2, not Figure 1). We have also adjusted the legend description for Figure 2 to provide clearer information.

Regarding the learning curves: we calculated them by averaging the fraction correct responses over blocks of 25 consecutive presentations of a single odor. These trials were then averaged across all stimuli within the set size (2 odors for set size = 2, 4 odors for set size = 4 from each session) and plotted with SEM for each group. We chose to bin the trials in blocks of 25 to reduce trial-by-trial variability and improve our ability to compare learning across set sizes, as the pseudorandom and interleaved presentation of odors makes trial-to-trial performance comparisons challenging. This method provides a clearer view of overall trends in performance when compared to the raw learning curve shown here.

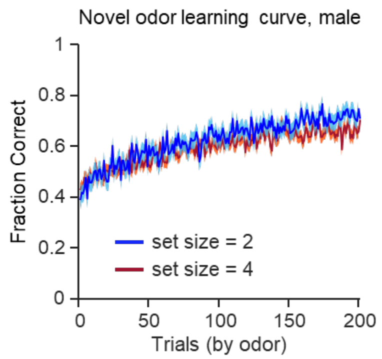

See below for our updated section in Materials & Methods:

“**Behavioral visualization and statistics**: In order to understand how mice learn to associate odor with rewarding choices at a high level, we first performed a series of behavioral analyses on the data using MATLAB (version 2018, 2021b, 2022b) and GraphPad Prism (version 9.1.1). We calculated learning curves (see Fig. 2A,D) by taking the mean fraction correct responses out of 25 presentations of a single odor. We then took the mean of all stimuli binned in 25 trials (2 odors for set size = 2 and 4 odors for set size = 4) and plotted the mean with SEM for all mice in the group. This allowed us to compare more directly across set sizes as all odors in a session have a pseudorandom and interleaved presentation. The learning curves presented here reflect the same sessions modeled (a maximum of 3 sessions per mouse per set size). To test if there was any difference between set sizes, we validated assumptions of normality and then ran a 2-way repeated measures ANOVA in GraphPad Prism with results reported in the main text. Next, we tested whether there was a difference in current trial performance (trial t) when the current stimulus was repeated or non-repeated in trial t-1 (see Fig. 2B,E). Starting at trial t+1 for each individual session for each animal, we took the result of trial t given t-1 (0 for incorrect or 1 for

correct) and placed the result in two new columns either for repeating or non-repeating stimuli. Then, we took the mean (fraction correct) performance for each individual animals' 3 sessions of set size = 2 and 3 sessions of set size = 4. Next we took the mean of all male or female mice and took the group mean and plotted each column for each set size with SEM. After checking assumptions for normality and variance using Anderson-Darling, D'Agostino and Pearson test, Shapiro-Wilk test, and Welsh's test, we used the appropriate comparison test (paired t-tests for within subjects and either unpaired t-tests or Mann-Whitney tests if not). Significance level was set to $p < 0.05$ for all statistical tests, and confidence intervals as well as effect sizes were reported where applicable. In Fig 2C,F the mean fraction correct for each session (including both set size = 2 and set size = 4) was plotted with individual animals connected by lines. To assess the impact of age on performance, separate mixed linear models were fitted for each sex using *statsmodels* package in Python. The models included age as the fixed effect predictor variable with additional fixed effects for session number and set size. Random effects (intercept and slope) were included for individual animal identity."

6) Figure 2B) reports some results that are not further discussed. How do the authors explain the difference in performance for non-repeated trials across-set sizes observed in males (and female as well though not significant)? Line 248-251: The authors' interpretation of this result is not clear at all.

Answer: Thank you for raising this concern. We have now clarified this point in our manuscript to better explain the difference in performance for non-repeated trials across set sizes. We intend to suggest the significant difference between set size 2 and set size 4 for repeated trials (2>4) and non-repeated trials (4>2) is due to the fact that a mouse using a one back strategy like S2=S4 is more likely to do well when stimuli are repeated more often in set size 2 and when non repeated stimuli have overlapping action choice in set size 4. This observation therefore launches our investigation of one-back strategies.

We now write:

"The weak set size effect in males favoring set size = 2 could be due to the fact that stimuli are repeated more often in set size 2. Notably, in non-repeated trials, fraction correct was higher in set size 4 compared to set size 2. (Fig. 2B: Mann-Whitney $U = 7507$, $p < 0.0001$). This could be because in set size 4, but not set size 2, the same action is correct for half the stimuli, even when the stimulus has changed. Thus, although we saw set size differences in males, this may be due to the success of repeating a choice rather than a signature of WM."

7) When comparing the learning curves between male and female mice, it appears that the set size effect goes in opposite directions between sex groups, and during different leaning periods (i.e. "early and "late" learning). In the early trials, although the effect is not significant, female mice seem to perform better in the test size = 4 condition. This is true even looking at figure 2B, in which the difference in performance between set size 4 and 2 looks greater in female then in male. I wonder if the overall lack of significance effects in the female sample is only due to statistical power considering the reduced sample size of female mice (n = 15).

Answer: Thank you for bringing this up. We agree that the relatively lower number of female animals (n = 15) could certainly contribute to lack of significant effects in some comparisons. However, n=15 is not

unusually low for mouse behavioral experiments, particularly for within-subject comparisons. We did not have the resources to increase the female sample size in this study in revision.

8) In general, the preliminary analysis is confusing and does not introduce the reader to the subsequent use of regressions and models. The standard way to report results in the RLWM (Collins et al., 2014, 2017; Collins & Frank, 2018; Collins & Frank, 2012) is as follows: average learning curve at each set size, effect of delay on performance (consistent with a time-based decay (forgetting)), and set size effect on early versus late learning. I suggest the authors to re-organize preliminarily analysis and behavioral results accordingly, even though this may emphasize the absence of a WM effect.

Answer: Thank you for your suggestion. We acknowledge that the standard practice in human RL+WM studies as outlined by Collins and colleagues (2012, 2018, 2020), emphasizes learning curves, the effect of delays (time-based forgetting), and set size differences between early and late learning phases. However, we believe that the way we have currently presented the data in mice is appropriate for highlighting the species-specific differences in behavior.

Our aim in the preliminary analysis is to demonstrate that mice do not show the traditional set size differences expected if they were using human-like WM processes. While we have included average learning curves for each set size (Panel A for males and D for females), the patterns in these curves indicate that the mice do not display the same WM-related effects seen in humans (e.g. increased performance earlier in lower set sizes). Instead, we observe a consistent "one-back" advantage (shown in second panels: B for males and E for females), particularly in male mice, across both set sizes. This is the key finding that we believe introduces the subsequent regression analyses and modeling, which further explore the impact of recent trial history rather than traditional WM effects. The final panels (C and F) show that learning performance across age doesn't change, again suggesting that these mice are not employing RL strategies similar to humans.

We believe that this approach sets up our additional analyses by emphasizing that the mice are behaving in ways that diverge from human RLWM models, supporting the absence of human-like WM effects. While reorganizing the analysis in line with human studies would indeed highlight the absence of these effects, we feel that our current presentation better leads into our modeling results, which show how mice may be employing short-term, trial-by-trial strategies instead.

9) The authors next used three logistic regressions to investigate the effect of long-term versus recent history on mice choice behavior. First, again the methods are poorly described. From line 119 to 126 the authors described the predictors for regression 1. Then they introduced regression 3 without explaining the meaning of predictors of regression 2. This forced me to go back to methods and results several times to understand how regression 2 and 3 differed, rendering it difficult to understand the logic behind the analysis.

Answer:
Thank you for drawing our attention to the regression analyses and the relative incompleteness of our regression descriptions. We initially performed a total of six regression models that included various combinations and interactions of repeated and rewarded trials. We had not included all the details of

our additional regressions in our initial draft; but, because of reviewers' suggestions, we have now placed a complete description of each into the methods, highlighted only regression 3 in the main text (Fig. 3, 4, and 5) with regression 2 in the supporting information (Fig. S4, S5, S6) and included a supplemental figure showing the results of an AIC comparison across all 6 regressions we tested (Fig. S1).

Below is a short description of our 6 regressions (now also included in Materials and Methods):

- Regression #1: correct/reward ~ intercept + correct history + 1 trial back + 2 trial back.
- Regression #2: correct/reward ~ intercept + correct history + main repeat + repeat-reward + norepeat-reward (where rewarded = 1, not rewarded = -1).
- Regression #3: correct/reward ~ intercept + correct history + main reward + reward-repeat + noreward-repeat (where repeated = 1, not repeated = -1).
- Regression #4: correct/reward ~ intercept + correct history+ repeat-reward + repeat-noreward + norepeat-reward + norepeat-noreward (where each interaction term is either 0 or 1).
- Regression #5: correct/reward ~ intercept + correct history + main repeat + main reward + reward*repeat.
- Regression #6: correct/reward ~ intercept + correct history + main reward + main repeat + repeat-reward + nrepeat-reward (where interaction term rewarded = 1, not rewarded = -1).

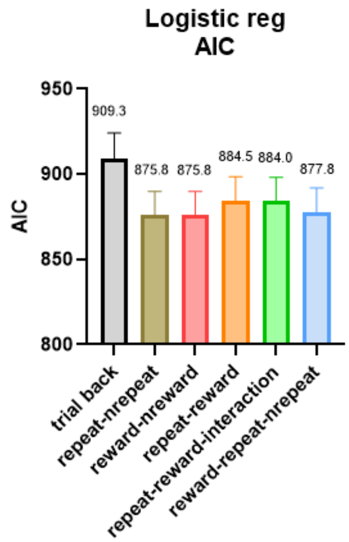

Model comparison: Using AIC comparison (as well as AICc, CAIC, and BIC), regression #2 and #3 are better models than regressions 1,4,5,6 (see included figure and now Supporting Information Figure 1 (Fig S1) in our manuscript).

Regression #2 and #3: In our winning regressions, we looked at the main effects of repeat or reward separately and also looked at how each main effect interacted with the other factor. For example, in regression #2 (correct/reward ~ intercept + repeat + repeat-reward + norepeat-reward), both rewarded and not rewarded were bidirectionally coded in the term repeat-reward and norepeat-reward, where rewarded = 1, not rewarded = -1, accounting for all possible interactions between repeat and reward. We separated regression #2 and #3 under 2 angles, where regression #2 examined repeat odor presentation as driving the main behavioral differences while effects of reward were captured in interaction terms. Regression #3 examined previous rewards while the effects of repeated odors were minor and captured in interaction terms. These are now discussed in our Materials and Methods section.

In modeling space, regression #2 and #3 contain terms that are interchangeable using linear combination computation, and are thus mathematically equivalent. Despite this, performing regressions separately allowed us to examine the results through different angles and to interpret effects of interactions accordingly.

We now discuss our regression model results more thoroughly with AIC comparison in new supplementary figures and information (Figure S1) and keep regression #3 alone in the main figures (Figures 3-5).

10) I do not understand what main effect “repeat” in regression is 2. Is this the same as 1 trial back in regression 1?

Answer: Yes, the "main effect repeat" in regression 2 is equivalent to the "1 trial back" parameter in regression 1. We used different terms to clarify the focus of each regression. Regression 1 aimed to capture working memory capacity by separating "1 trial back" and "2 trials back" specifically. Regression 2, on the other hand, consolidated the focus on whether the current trial repeated the previous trial’s stimulus. Regression 2 is now in the supplemental materials and regression 3 is reported in the main text.

11a) I suggest using a hierarchical approach (i.e., stepwise regression) to select which predictors to include in the model (I am skeptical that the interaction terms improve the model’s performance significantly). Further the authors should compare the performance of the three models via AIC/ BIC criteria.

Answer: Thank you for the suggestion regarding a hierarchical approach. Instead of using a stepwise regression, which can suffer from multicollinearity and reduced statistical power, we opted to develop and test multiple logistic regression models based on different hypotheses and interactions between factors. We then used AIC, BIC, AICc, and CAIC to select the best-fitting models, which are now included in the supplementary figures (Figure S1). This approach allowed us to avoid potential pitfalls of hierarchical regression while comparing multiple models objectively. As mentioned, regressions 2 and 3 were found to be superior using AIC comparisons.

11b) Line 137: why is the reward-repeat regressor attributed mostly to a WM-like process? A simple RL mechanism (i.e., value updating) can account for the positive effect of previous rewarded choice on current trial.

Answer: In regression 3, the correct history term captures the accumulating reward information and value updating that is core to reinforcement learning (RL) processes. The reward-repeat regressor takes into account not just whether an animal received a reward, which you correctly associate with a rudimentary RL mechanism such as value updating, but also whether the stimulus on the current trial is the same as the previous trial when the animal made the correct choice (thus receiving a reward). A correct choice in this condition could rely on the animal holding not just the reward, but the stimulus-action contingency in memory. Furthermore, we assume that the contribution of RL is captured by the cumulative reward history regressor instead; the reward repeat regressor captures variance that is not already captured by the cumulative reward history regressor, and thus is likely to correspond to a different process. However, we agree that this is not guaranteed; computational modeling provides a stronger interpretation than the regressions.

12) Line 294-323: it is difficult to understand the message that the authors are trying to convey. line 305-306 the authors attribute this effect to a sort of WM process ("as expected if mice can maintain and leverage the correct information", see comment 11 on the interpretation of regressors as indicative of WM). Then, they claim that because of the negative effect of reward on non-repeat trials, it might be possible that male mice might be sticking to the previous choice if that one was rewarded. They used the difference in magnitude observed between "repeat-reward" and "non-repeat reward" to conclude that both a sticking tendency and the use of previous trial information can account for mice behavior. This result might simply indicate that the mice are learning the task, and consequently, the tendency to repeat previous choices regardless of the current information diminishes as the mice learn to use the reward prediction error to guide their choices. This is also valid considering that the authors are analyzing only the first 200 repetitions of each session. In other word this difference in magnitude between the 2 coefficients should increase as a function of time (i.e. trials within sessions or even sessions along days)

Answer: Thank you for your comments, and we acknowledge that this section may not have been as clear as it could be. We agree that the result you highlighted—where we observe a difference in magnitude between "repeat-reward" and "non-repeat reward"—could indeed reflect ongoing learning rather than purely working memory (WM) processes.

To clarify, our intention was to point out that both WM-like processes and reinforcement learning (RL) may be at play. Specifically, the "repeat-reward" coefficient suggests that when male mice receive a reward for a repeated stimulus, they are able to maintain and leverage that information for future decisions, aligning with WM-like behavior. This is the same for "norepeat-reward." Both of these regressors are capturing a fast paced process (one-trial back learning) instead of RL, which is captured by our "correct history" parameter.

We entirely agree that these processes are interconnected and are often difficult to parse, especially in a regression analysis. Thus our regressions (and later our modeling) attempts to do so by separating the slow, incremental process of RL ("correct history" and then our alpha learning rate and noise parameter beta in modeling) and a faster, one-trial back process that is akin to WM (all of our one-trial back regressors and our strategy parameters in the modeling phase). A correct choice in this condition could rely on the animal holding not just the reward, but the stimulus-action contingency in memory.

13) 350-353: clearly the meaning of the regressors in regression 2 still escapes me. Which regressor of figure 3B the authors are referring to? "norepeat-reward"????. Is this coefficient capturing the tendency of mice to repeat previous rewarded choices independently of the cue, and thus impacting negatively the performance? If this is the case, do the authors agree that an alternative, valid conclusion can be that as mice age they reduce the tendency to alternate responses (i.e. less random exploration see for example Nussenbaum & Hartely 2019)?

Answer: Yes, we were describing the "norepeat-reward" regressor, a regressor that describes the likelihood of a mouse making the correct choice following a reward when there is a different odor cue

on trial *t* than on trial *t-1*. A negative trend implies that the mouse is performing a win-stay behavior, which can also be reflected in your hypothesis that mice reduce the tendency to alternate responses. We also understand that the two similar regressions (regression #2 and regression #3) were redundant and their interpretation less clear. As described above, we decided to leave regression #3 in the main text and moved regression #2 to the supporting information.

14) Line 384: this might be true also for regressions coefficients

Answer: Thanks for bringing this to our attention. This is indeed something that is useful to check. Below, see our correlation analysis for regression #3 (the regression tied for lowest AIC/BIC that is now our main regression that we include in the main text). The regression intercept and the correct history parameter (here described as reg_reward_rl) as well as the main effect of reward and the interaction between reward and repeat are both the most correlated. The latter we could expect since both reward main and reward_repeat are drawing on the same one-back rewarded information. As for intercept and correct-history, this is a bit more unexpected and instead suggests that there may be something else driving animal's performance that is missed in our current regression. There is also a negative correlation between no_reward_repeat and both correct history and the main effect of reward. This also makes sense given that there are always two outcome options (reward or no reward) which oppose each other. Despite some of these correlations, we tested multicollinearity with variance impact factors (VIF) as reported in our Materials and Methods and removed any points outside of 3 standard deviations of the mean (z-scored) before we visualized or analyzed any data. This is now reported in our Materials and Methods and the below figure is now in Supplemental Figure 1B.

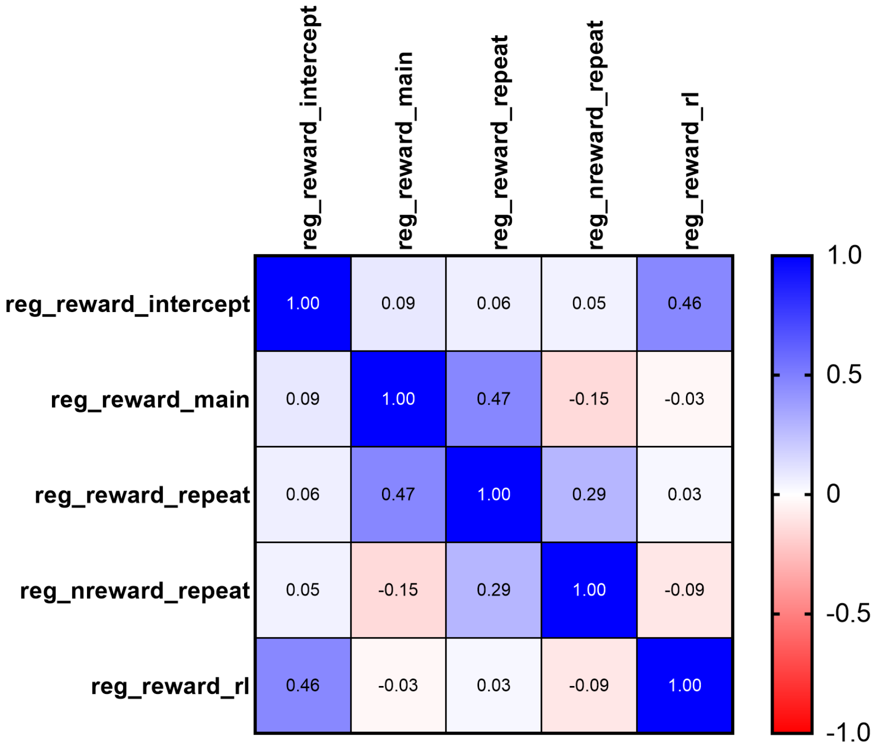

15) Considering the quite high number of parameters I wonder whether the model does not suffer from overfitting the behavioral data. I recommend running a cross-validation analysis to test for overfitting.

Answer: We appreciate the suggestion to run a cross-validation analysis, but we don't believe that this is necessary for the following reasons:

- While cross-validation should be done to avoid overfitting when it is feasible, it is not feasible in our case since we fit per session and thus cannot isolate independent subcomponents within a session (due to the session data being a single learning trajectory with sequential dependencies).
- We can verify that we are not overfitting during parameter identification with parameter recovery analysis (see our new Fig. S2) which is very successful.
- Any overfitting in model comparison is handled by penalization criteria (AIC) in model comparison (see Fig. 6).
- Lastly, the number of parameters is standard for this kind of modeling, especially given the large amount of data per session.

From our Materials & Methods:
"We used standard maximum likelihood estimation to estimate best-fitting parameters (Wilson & Collins, 2019). While we considered using more state-of-the art methods such as *maximum a posteriori* or hierarchical Bayesian modeling, we found this unnecessary in this case, as the large number of trials afforded very good parameter identification (tested through a generate and recover procedure) with the simpler method, with no need for the additional assumptions that more advanced methods require (e.g. priors). We fit separately each individual and session, obtaining a set of best fit parameters for each individual and session."

16) Figure S3 should be reported in the main text.

Answer: Old Figure S3 is now combined with Figure 3 for regression #3 and is in the main text with regression #1 (set size = 2 and set size = 4). We appreciate the suggestion.

17) Figure S2: it is difficult to guess how well the model captures data variability considering the authors plotted the entire course of choices in sessions where set size=4? The authors should generate a figure that compares model and the behavior as reported in fig. 1.

Answer: Thank you for this suggestion as well. We now binned the data in the same way as we did in Figure 2A,D and now show in the supplemental figure the results from simulated data for set size =2 and set size = 4 for both male and female animals. The new figure is now Fig. S3.

18) The discussion is still raw and does not provide a clear take-home message. To summarize: The results indicate that in this task, mice employ a RL process. Adding the one-back component as an additive to the RL process might not be necessary, as it may already be part of it. As discussed by the authors (line 497), there is the possibility that, given the nature of the task, mice may have used other learning strategies less reliant on WM. However, the authors do not describe what these strategies are and which aspect of the task the authors believe most affected the implication of WM.

Answer: We believe there may be a misunderstanding about the take-home at the end of the discussion. We intend the take-home to be that mice use RL and one back strategies, but NOT canonical WM to solve the task. We do not examine the reasons WM is absent beyond species differences.

Rather, in the closing of the discussion we point out that other labs have also recently suggested mice solve tasks with different strategies that alternate for example from engaged to disengaged when overtrained in a decision making task (Ashwood et al., 2022). This is meant to be a 'nod' toward other recent work related to our own.

"Finally, our data add to an ongoing discussion of the multiple components contributing to laboratory task behavior in rodents. Other recent papers have argued that mice do not only rely on single strategies, but use a wide repertoire during decision making (Ashwood et al. 2022)."

We then seek to explain how our work adds to this developing area of discovery. We suggest our model is different because it can account for use of RL and choice strategies together and not just during decision making but during learning.

"Here, we also consider how multiple strategies contribute to decision making in the context of learning, but instead of assuming that animals switch between them over the course of the experiment, we show how they can be used concomitantly throughout a learning session. Thus our approach differs from others (Bolkan et al., 2022; Ashwood et al., 2022; Le et al., 2023) by looking at how multiple parallel processes contribute to behavior, allowing for processes that could be accumulative or short-term, rather than multiple strategies supported by the same process."

We hope this clarifies any misunderstanding. If there is a better way to phrase the discussion to make this more clear we would appreciate guidance. We also apologize if we misunderstand what is being asked in this comment.

Line 507: The authors should have data available to examine the relationship between mice's weight and the value of parameter S1==S4, corroborating their assumptions. Additionally, the authors should discuss the possible interactions between age, sex, and performance considering the complex interaction between sex hormones and learning abilities reported in numerous rodents studies (see, for example dalla & Shors 2009, Orsini et al., 2020; Piekarski et al., 2017).

Answer: We appreciate the suggestions for making our paper and discussion section stronger.
We are quite upset to find that we did not properly preserve the weight data for our original behavioral data, therefore we could not further evaluate our speculation about weight and changes in male behavior with age. We have now removed the speculation due to other reviewer comments.

To test if there was a role for testosterone at puberty and the gain in S2=S4 in males, we have now added data from an experimental group of young adult males who underwent prepubertal gonadectomy or sham control surgery (n = 6 for GDX and n = 5 for sham) (Fig. 10 and S7). We found no significant differences in learning and almost all model parameters between GDX and sham groups, except for a significant difference in the S3 strategy parameter. Importantly, alpha learning rate,

softmax beta, and one back strategy S2=S4 was comparable. We speculate that the difference in S3 was driven by an effect of surgery in sham rather than GDX due to differences in sham when comparing to data from intact mice (See Fig. 10).

Overall, we have since edited the discussion to be more neutral. We now write in discussion:
“These data suggest a rise of testosterone at puberty did not play a causal role in any of the changes seen in one-back strategies and was not needed to support basic performance in males. Future studies might turn to other biological changes that may be sex-specific but decoupled from puberty to understand what drives changes in strategy use in males with development.”

19) line 529: I would appreciate an integration with the current literature on developmental changes in RL and WM capacity observed in rodents that highlights the possibility that other tasks choices (see for examples Afshar et al., 2020)) may illuminated developmental changes in those processes. The “early emergence “ of an adult like function seems like a speculative assertion considering the limited available research on learning development in mice

Answer: Thank you for bringing Afshar et al. to our attention. We now report this finding and note how it contrasts with our data but differs in that involves reversal learning. We have expanded our discussion of the development of cognition in mice and now cite this paper. To shore up our speculation about the lack of age difference with more evidence, we also now consider that repeated odor-action learning in our current manuscript has some parallels with studies of intradimensional shifts (IDS) in adolescent rodents often using odor cues to inform learned choice of one of 2 pots. Here again, in IDS learning age differences were not apparent, consistent with our data.

Text from our modified discussion: “Our experiments also allowed us to examine mouse behavior across adolescent development. We did not observe any significant developmental changes in basic performance metrics, learning rate alpha, and the softmax inverse temperature parameter beta, with age. The lack of change in the RL learning rate metric and the softmax noise parameter was somewhat surprising in light of the human literature and some rodent literature, but is perhaps consistent with some existing studies of repeated discrimination learning in rodents.

Briefly, in human participants, the softmax beta parameter has been shown to (most) consistently decrease across development in humans and the alpha learning rate parameter has (less consistently) been shown to increase with adolescent age in some, but not all, task contexts (reviewed Nussenbaum et al., 2019; Eckstein et al., 2022; Wilbrecht & Davidow, 2024). Increases in RL learning rate with age have also been reported in rodents, but only for reversal learning, not initial discrimination learning (Afshar et al., 2020).

In discrimination learning without reversal or before reversal, the age performance relationship can be flat or sometimes, adolescent rodents can learn even faster than adults (Afshar et al., 2020; Konstantoudaki et al., 2018). In studies of attentional set shifting (ASST) tasks, where rodents learn a series of two choice odor-action or texture-action contingencies, adolescent and adult rats learn about

new cues at a similar rate when the same modality is reinforced (intradimensional shift, IDS) but reach criterion more slowly in when the modality is changes (extradimensional shift, EDS)(Newman et al., 2011; Afshar et al., 2020). In mice, results are more mixed with some showing no age differences in IDS and EDS performance (Johnson et al., 2011), while others find adolescent mice underperform adult mice on an initial IDS or EDS but by the second IDS session adolescent learning is comparable to adults (Shepard et al., 2017). (Note that no computational modeling was reported in these set shifting studies). In the current study, the fact that we present multiple pairs of novel odorants to be associated with the same two actions and without any training on reversal makes our data most comparable to the IDS performance metric. In this light, a lack of change in fraction correct and RL learning rate in mice in the RL+WM task across development can be seen to be consistent with previous findings. Based on the needs of rodents in the wild, we speculate that learning systems may need to be intact just after weaning to support dispersal and the transition to independence (Lin et al., 2022).

While alpha learning rate and the softmax beta parameters did not change significantly with development in either males or females, we did find a consistently significant and positive age-related relationship in male animals in the one-back choice strategy S2=S4: Stimulus Insensitive Win-Stay and an age and experience dependent reduction in S1 and S3 choice strategies after negative outcomes. These relationships were not significant in female mice. To test if these changes in males were dependent on developmental increases in testosterone, we examined task performance and model parameters in a cohort of mice who had undergone pre-pubertal gonadectomy or a sham surgery. We found that at young adult ages in GDX and sham groups, there were no differences in learning curves or model parameters, except for S3. Here the sham group was lower than seen in intact mice of the same age, suggesting a possible effect of surgery. These data suggest a rise of testosterone at puberty did not play a causal role in any of the changes seen in one back strategies and was not needed to support basic performance in males. Future work could investigate how longitudinal weight gain might relate to longitudinal gain in strategy use, but loss of individual weight data prevented us from examining this idea further."

Also regarding adolescent development, we hope the reviewer will find the study enriched by adding a study of the effects of prepubertal gonadectomy (Fig. 10).
