# Supplementary material for: Adolescent and adult mice use both incremental reinforcement learning and short term memory when learning concurrent stimulus-action associations
Source: PLoS Comput Biol. 2024 Dec 23;20(12):e1012667. doi: 10.1371/journal.pcbi.1012667 (PMC11706416; doi:10.1371/journal.pcbi.1012667)
Supplement: Attachment — Submitted filename: ReviewPLOSW.docx [file pcbi.1012667.s009.docx]

1) The authors need to better justify their task design. Why was each stimulus uniquely associated with one action? This choice may have led the mice to infer the correct action for certain odors based on knowledge of the correct action for other odors, thereby potentially reducing the implementation of both RL and WM This is a possibility also considering that before being introduced to novel odor sets mice started the session with the A/B task to which they were overtrained. In other words, the presentation of the AB task could have served as an initial recall of the task rule (Odor X = Left,Odor Y the opposite), increasing the likelihood that the mouse would utilize a “by-elimination” strategy in the subsequent block.

2) Further it is not specified how many new odors the author used in total and how did they manage block presentation ordering. All this information should be in the Materials and Methods section.

3) The rationale behind the inclusion of the readiness check should be better justified, considering that the motivational aspect could have been controlled post-hoc discarding sessions in which mice did not reach the minimum number of trials performed to be considered engaged in the task.

4) Material and Methods:

line 95-112 : I strongly recommend to re-organize the entire section. Considering the difficulty, I experienced in understanding what was done in figure 1, I think this section should include a description of all the dependent variables that the author analyzed to preliminarily inspect behavior (and how they were computed), and the rational for the analysis.

5) Figure 1 A: It seems that the authors present a learning curve where consecutive trials over the first 200 presentation for each stimulus were divided into 8 bins, and performance was averaged within each bin, is the interpretation correct?

I cannot find an explicit reference in the text regarding how the learning curve was calculated, and consequently, how the analysis for evaluating the set size effect was conducted. The set size effect in male mice appears to be so weak that I wonder if it remains significant across the entire curve (i.e., non-binned). If the data were binned the authors should specificized why and how. The authors should show the raw learning curve at least (starting from repetition 1).

6) Figure 2B) reports some results that are not further discussed. How do the authors explain the difference in performance for non-repeated trials across-set sizes observed in males (and female as well though not significant)? Line 248-251: The authors' interpretation of this result is not clear at all.

7) When comparing the learning curves between male and female mice, it appears that the set size effect goes in opposite directions between sex groups, and during different leaning periods (i.e. “early and “late” learning). In the early trials, although the effect is not significant, female mice seem to perform better in the test size = 4 condition. This is true even looking at figure 2B, in which the difference in performance between set size 4 and 2 looks greater in female then in male. I wonder if the overall lack of significance effects in the female sample is only due to statistical power considering the reduced sample size of female mice (n = 15).

8) In general, the preliminary analysis is confusing and does not introduce the reader to the subsequent use of regressions and models. The standard way to report results in the RLWM (Collins et al., [2014](https://link.springer.com/article/10.3758/s13423-020-01774-z#ref-CR9), [2017](https://link.springer.com/article/10.3758/s13423-020-01774-z#ref-CR10); Collins & Frank, [2018](https://link.springer.com/article/10.3758/s13423-020-01774-z#ref-CR13); Collins & Frank, [2012](https://link.springer.com/article/10.3758/s13423-020-01774-z#ref-CR8)) is as follows: average learning curve at each set size, effect of delay on performance (consistent with a time-based decay (forgetting)), and set size effect on early versus late learning. I suggest the authors to re-organize preliminarily analysis and behavioral results accordingly, even though this may emphasize the absence of a WM effect.

9) The authors next used three logistic regressions to investigate the effect of long-term versus recent history on mice choice behavior. First, again the methods are poorly described. From line 119 to 126 the authors described the predictors for regression 1. Then they introduced regression 3 without explaining the meaning of predictors of regression 2. This forced me to go back to methods and results several times to understand how regression 2 and 3 differed, rendering it difficult to understand the logic behind the analysis.

10) I do not understand what main effect “repeat” in regression is 2. Is this the same as 1 trial back in regression 1?

11) I suggest using a hierarchical approach (i.e., stepwise regression) to select which predictors to include in the model (I am skeptical that the interaction terms improve the model’s performance significantly). Further the authors should compare the performance of the three models via AIC/ BIC criteria.

11) Line 137: why is the reward-repeat regressor attributed mostly to a WM-like process? A simple RL mechanism (i.e., value updating) can account for the positive effect of previous rewarded choice on current trial.

12) Line 294-323: it is difficult to understand the message that the authors are trying to convey. line 305-306 the authors attribute this effect to a sort of WM process (“as expected if mice can maintain and leverage the correct information”, see comment 11 on the interpretation of regressors as indicative of WM). Then, they claim that because of the negative effect of reward on non-repeat trials, it might be possible that male mice might be sticking to the previous choice if that one was rewarded. They used the difference in magnitude observed between “repeat-reward” and “non-repeat reward” to conclude that both a sticking tendency and the use of previous trial information can account for mice behavior. This result might simply indicate that the mice are learning the task, and consequently, the tendency to repeat previous choices regardless of the current information diminishes as the mice learn to use the reward prediction error to guide their choices. This is also valid considering that the authors are analyzing only the first 200 repetitions of each session. In other word this difference in magnitude between the 2 coefficients should increase as a function of time (i.e. trials within sessions or even sessions along days)

13) 350-353: clearly the meaning of the regressors in regression 2 still escapes me. Which regressor of figure 3B the authors are referring to? “norepeat-reward”????. Is this coefficient capturing the tendency of mice to repeat previous rewarded choices independently of the cue, and thus impacting negatively the performance? If this is the case, do the authors agree that an alternative, valid conclusion can be that as mice age they reduce the tendency to alternate responses (i.e. less random exploration see for example Nussenbaum & Hartely 2019)?

14) Line 384: this might be true also for regressions coefficients

15) Considering the quite high number of parameters I wonder whether the model does not suffer from overfitting the behavioral data. I recommend running a cross-validation analysis to test for overfitting.

16) Figure S3 should be reported in the main text.

17) Figure S2: it is difficult to guess how well the model captures data variability considering the authors plotted the entire course of choices in sessions where set size=4? The authors should generate a figure that compares model and the behavior as reported in fig. 1.

18) The discussion is still raw and does not provide a clear take-home message. To summarize: The results indicate that in this task, mice employ a RL process. Adding the one-back component as an additive to the RL process might not be necessary, as it may already be part of it. As discussed by the authors (line 497), there is the possibility that, given the nature of the task, mice may have used other learning strategies less reliant on WM. However, the authors do not describe what these strategies are and which aspect of the task the authors believe most affected the implication of WM.

Line 507: The authors should have data available to examine the relationship between mice's weight and the value of parameter S1==S4, corroborating their assumptions. Additionally, the authors should discuss the possible interactions between age, sex, and performance considering the complex interaction between sex hormones and learning abilities reported in numerous rodents studies (see, for example dalla & Shors 2009, Orsini et al., 2020; Piekarski et al., 2017 ).

19) line 529: I would appreciate an integration with the current literature on developmental changes in RL and WM capacity observed in rodents that highlights the possibility that other tasks choices (see for examples Afshar et al., 2020)) may illuminated developmental changes in those processes. The “early emergence “ of an adult like function seems like a speculative assertion considering the limited available research on learning development in mice
